# Water, sanitation, and hygiene insecurity and disease prevention behaviors during the COVID-19 pandemic in low-income neighborhoods of Beira, Mozambique

**DOI:** 10.1371/journal.pone.0310490

**Published:** 2024-11-21

**Authors:** Rebecca S. Kann, Jedidiah S. Snyder, Courtney Victor, Zaida A. Cumbe, Joshua V. Garn, Sandy McGunegill, Rassul Nalá, Matthew C. Freeman, Karen Levy

**Affiliations:** 1 Department of Environmental and Occupational Health Sciences, School of Public Health, University of Washington, Seattle, Washington, United States of America; 2 Gangarosa Department of Environmental Health, Rollins School of Public Health, Emory University, Atlanta, Georgia, United States of America; 3 WE Consult, Maputo, Mozambique; 4 Department of Epidemiology, Biostatistics, and Environmental Health, School of Public Health, University of Nevada, Reno, Nevada, United Sates of America; 5 INS – Instituto Nacional de Saúde, Ministério de Saúde, Maputo, República de Moçambique; Arizona State University, UNITED STATES OF AMERICA

## Abstract

**Background:**

Inadequate water, sanitation, and hygiene (WASH) are important drivers of the global burden of disease, and their impact is exacerbated during outbreaks. Directives to practice handwashing and physical distancing may be impractical for people that have limited access to WASH resources. In this study, which took place during the acute phase of the COVID-19 pandemic, we explore the relationship between control measures for global health crises and water, sanitation, and hygiene insecurity, with implications for other infectious diseases and future health emergencies.

**Methods:**

We investigated the relationship between WASH-related factors and disease prevention behaviors (handwashing, physical distancing, and masking), and the role of demographic characteristics and risk perceptions in influencing these relationships in low-income neighborhoods of Beira, Mozambique during the COVID-19 pandemic. We utilized data collected from 1,762 randomly selected households during a population-based survey. We fit multivariable logistic regression models to examine the associations between various WASH factors and disease prevention outcomes of interest, adjusting for individual- and household-level demographic characteristics and risk perceptions.

**Results:**

Over 98% of people had access to improved drinking water and over 80% of people had access to improved sanitation facilities. There was a high level of reported adherence to handwashing (95.5%) and physical distancing (91.7%) practices during the COVID-19 pandemic. There was a lower odds of reported handwashing [aOR = 0.89; 95% CI: 0.81, 0.98] and physical distancing [0.85 (0.80, 0.92)] among respondents who had higher levels of water insecurity. Respondents that had a water source in their dwelling had a higher odds of reporting of physical distancing [2.03 (1.22, 3.41)] compared to people that had to leave their household to access water. There was a higher odds of reported handwashing and physical distancing among respondents who had their own sanitation facility, compared to a shared one [handwashing: 2.77 (1.35, 5.82); distancing: 1.61 (0.95, 2.73)], and those that had a sanitation facility inside their compound compared to outside their compound [handwashing: 2.11 (0.75, 5.71); distancing: 1.50 (0.65, 3.36)]. Respondents with a basic handwashing station, compared to no facility or a limited facility, had a higher odds of reported handwashing [4.45 (2.37, 8.65)], and those that had a connected handwashing station, compared to an unconnected handwashing station, had a higher odds of reporting handwashing and physical distancing [handwashing: 2.13 (0.68, 8.54); distancing: 1.77 (0.77, 4.53)].

**Conclusions:**

Despite a high level of knowledge about the risks posed by COVID-19 and understanding of the benefits of handwashing and physical distancing, limitations in access to water, sanitation, and hygiene infrastructure acted as a barrier to people practicing disease prevention behaviors during the COVID-19 pandemic.

## 1. Introduction

Water, sanitation, and hygiene (WASH) inequities are important contributors to the global burden of disease [1]. In response to the COVID-19 pandemic, public health strategies emphasized handwashing, alongside directives to self-quarantine and practice physical distancing, as strategies to slow the spread of SARS-CoV-2 transmission [2]. In low- and middle-income countries (LMIC), WASH insecurity can create challenges for practicing handwashing and physical distancing in response to an outbreak. Over 2 billion people lack access to household handwashing facilities, limiting their ability to practice proper handwashing behaviors [3, 4]. Additionally, many people rely on shared WASH facilities, which reduces the ability to practice physical distancing [4–6]. Understanding the role of these different WASH factors as determinants of handwashing and physical distancing behavior in the context of an active pandemic can provide important information for improving control measures during future global health crises [7].

Previous research exploring behavioral determinants of disease prevention has shown mixed evidence of the specific role of individual- and household-level factors, and such evidence is especially limited in the context of outbreaks or crises [8]. With a renewed focus on handwashing and physical distancing during the COVID-19 pandemic, several studies have found mixed evidence for the role of demographic characteristics and knowledge, attitudes, and practices (KAP) of disease prevention measures within the context of the active pandemic. Even when knowledge of the pandemic and risk factors are high, cultural norms, varying perceptions of the effectiveness of control measures, and demographic factors including age, education level, and occupation, influence people’s decisions and abilities to practice disease prevention strategies [9–12]. Public health directives that promote handwashing and physical distancing must consider how individual- and household-level factors, as well as perceptions of risk, impact decisions to practices disease prevention behaviors.

Access to improved WASH facilities may also act as a determinant of disease prevention behaviors [1, 4, 8, 13]. Handwashing is a key prevention strategy implemented to reduce transmission of both enteric and respiratory infectious diseases [3, 14–17]. Effective handwashing requires access to facilities that enable hygiene behaviors, which includes access to clean water and soap [6, 8]. However, as of 2019, approximately 26% of the global population, and over 50% of people in sub-Saharan Africa and Oceana, lacked access to handwashing facilities with soap and water [3]. Access to improved WASH resources is also important for enabling physical distancing practices. Physical distancing may not be possible for WASH insecure households that rely on shared water, sanitation, and handwashing resources [5, 18, 19]. Since the height of the COVID-19 pandemic, several studies have explored the ways in which WASH access, and specifically water access, influenced disease prevention behaviors [20–24]. Specifically, water demand in LMICs increased during the pandemic for use in handwashing, and water insecurity hindered people’s ability to adhere to COVID-19 protective measures [22, 23]. Few studies, however, have extended this analysis to explore the role of sanitation and hygiene access in influencing disease prevention behaviors.

Mozambique has historically been faced with high levels of WASH insecurity [25], an important driver of the burden of disease, and these issues were exacerbated by the onset of the COVID-19 pandemic [26, 27]. The first COVID-19 cases were detected in Mozambique on March 22, 2020. As of April 2023, there have been 233,214 total confirmed cases of COVID-19 (cumulative) and 2,242 total deaths attributable to COVID-19 in Mozambique [28], which is comparable, although slightly lower, than neighboring countries [29]. Despite findings suggesting high knowledge about COVID-19 and strong adherence to preventative measures in Mozambique [30, 31], COVID-19 has had a significant effect on the population in Mozambique. Mozambique’s response to the pandemic was complicated by several preceding humanitarian crises, including cyclones Idai and Kenneth and a subsequent cholera outbreak prompted by mass displacement of the cyclone-impacted population to shelters [26, 32]. Prior to the cyclones, WASH access across Mozambique was already limited, especially in rural areas [25]. As of 2020, approximately 63% of Mozambique residents had access to at least basic water sources and 37% had access to at least basic sanitation services [25]. Following the cyclones, WASH services in Mozambique were impacted by the large-scale damage of infrastructure, but also benefited from broad investments in improving WASH access by multiple non-governmental and governmental sources [26, 32].

Given the high levels of existing WASH insecurity and rapid shifts in infrastructure prior to the start of the COVID-19 pandemic, we aimed to understand what relationships exist between WASH factors, risk perceptions, and demographic characteristics and disease prevention behaviors in response to the COVID-19 pandemic. We expand on previous studies of determinants of disease prevention behaviors and assess the role of WASH insecurity, alongside other known determinants of behavior, within the context of a low-income setting and an active pandemic. Specifically, we ask the question: what is the relative importance of WASH, compared to risk perceptions and demographics factors, in influencing decisions to practice disease prevention behaviors, including handwashing and physical distancing? Findings from this study will be generalizable to other disease outbreak events in low-income settings by addressing gaps in evidence on determinants of handwashing behavior and providing insight into the complexity of the role of water, sanitation, and hygiene insecurity in adherence to control measures for global health crises.

## 2. Methods

### 2.1. Study setting and design

Data were collected as part of formative research for the PAASIM study (*Pesquisa Sobre o Acesso à Água e a Saúde Infantil em Moçambique—Research on Access to Water and Children’s Health in Mozambique*) [33] among residents of informal neighborhoods in Beira, Mozambique. Beira is the second largest city in Mozambique (population ~530,000) [34], which serves as a gateway for both the central interior portion of the country and a trade corridor to neighboring land-locked nations. The center of Beira is bordered by unplanned, informal settlements inhabited by over 300,000 low-income residents [33].

There were two rounds of data collection–one in 2019 and one in 2020. The primary analysis utilizes data collected in 2020 to quantify associations between WASH factors and disease prevention behaviors within the context of the acute phase of the COVID-19 pandemic. Data collected in 2019 was used to summarize changes in water sources leading up to the start of the COVID-19 pandemic, as presented in the Supplemental Materials (S1 Table).

A set of sub-neighborhoods for the survey were selected within the city center, primarily based on their characteristics as containing low-income, high-density, peri-urban housing, which we are examining as a part of the PAASIM study. Sub-neighborhood boundaries were delineated along natural boundaries such as roads or waterways. The number of households and population density of each sub-neighborhood was approximated using Google Earth satellite imagery using a random grid approach (see [35] for complete details). Household density estimates were used to proportionally sample households, where the probability of a household being selected into the study was proportional to the household density of the neighborhood. Enumerators used an interactive map of sub-neighborhoods to select a grid of their assigned area to begin sampling. Enumerators then used a random number generator to select a starting point among the first 19 houses and then systematically sampled every 19th household until all households had been counted in the sub-neighborhood, to provide approximately a 5% proportional sample.

### 2.2. Data collection

**Fig 1:**
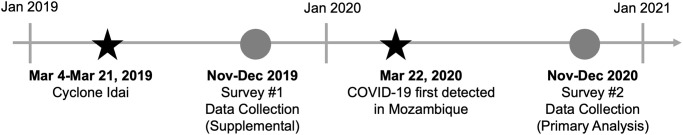
Timeline for data collection and influential events affecting study area.

The recruitment period and two rounds of surveys were conducted from November 16 to December 23, 2019 (eight months after Cyclone Idai) and November 7 to December 23, 2020 (eight months after COVID-19 was first detected in Mozambique) (Fig 1). Recruitment took place within household compounds. Enumerators contacted adult members of the household that were over the age of 18 and asked the respondent if s/he consented to participate in the study. The survey consisted of several modules, including recruitment, questions regarding household demographics, assets and wealth indicators, access to water and sanitation, and satisfaction with water service. In the 2020 survey, respondents were asked to provide information about access to handwashing facilities, self-reported COVID-19 prevention measures (handwashing, physical distancing, mask wearing) and perceptions towards COVID-19, handwashing, and physical distancing practices.

The primary analysis for this manuscript uses only the data from the 2020 survey. A supplemental analysis assessed longitudinal changes in WASH access between the 2019 and 2020 survey to add context about WASH factors to the primary analysis. Results from this analysis of changes in WASH between the two surveys can be found in the S1 Table.

Surveys were administered by enumerators electronically on password-protected mobile tablets equipped with the Open Data Kit (ODK) Collect app [36]. Submitted data were exported daily to a secure server to ensure data quality (e.g., quality assurance using geocoded data to ensure households were within study area boundaries and spot checks to assess for missing survey data).

### 2.3. Variables

#### 2.3.1. Demographic characteristics

We collected data on the sex, education level, and maternal status of the respondent as well as household-level characteristics, including the number of children under 5 years of age living in the household, presence of a pregnant woman, and household size. Education responses were analyzed using a 3-level ordinal variable: received no education, received up to primary education, or received higher education. The number of members in a household, or household size, was categorized using a 3-level ordinal variable: 3 or fewer people, 4 to 7 people, and over 7 people.

We assessed socioeconomic status using the Simple Poverty Scorecard^®^ Poverty-Assessment Tool Mozambique [37], where respondents answered ten standardized questions, including questions on household size, materials, and assets. Each question’s answer choices correspond with a point total, and points are summed over all ten questions into a poverty score. We use this poverty score to compare consumption of assets across different households, both using it as a 5-point continuous score and categorizing it into quartiles, with wealthier households having higher scores.

#### 2.3.2. WASH factors

Indicators of the quality of drinking water source, sanitation facility, and handwashing station were based on the definitions from WHO/UNICEF Joint Monitoring Programme (JMP) for Water Supply, Sanitation, and Hygiene [38]. Respondents were asked to provide information on the main source of drinking water for members of the household, the main type of sanitation their household used, and the main handwashing station. All variables using JMP definitions of WASH quality were evaluated on a binary scale (improved vs. not improved drinking water source, improved vs. not improved sanitation facility, basic vs. not basic handwashing station). Households were also asked whether they shared their main water source and their main sanitation facility with people outside their household and if their main handwashing station was fixed or mobile. Finally, water insecurity was quantified using the validated four-item Household Water Insecurity Experiences (HWISE) scale [39], and was included in the analysis on a continuous scale (0–12), where higher values indicate higher levels of water insecurity. These WASH factors were hypothesized to be important determinants of COVID-19 prevention behaviors due to their necessity for performing handwashing and the reduced capacity for physical distancing when WASH services are shared or were not easily accessible, as well as the impacts on various psychosocial and behavioral factors.

Additional facets of water insecurity including questions related to water availability, accessibility, affordability and satisfaction [40] were evaluated in supplementary analyses to assess changes in WASH access leading up to and during the time of the primary analysis (S1 Table). Details on these additional variables can be found in the supplemental materials. These additional WASH variables were not included in the regression models for the primary analysis because they were considered to exist along the causal pathway for the main exposure and outcome variables of interest.

#### 2.3.3. Perceptions of risks and health behaviors

Respondents were asked to respond on a 5-point Likert scale (strongly disagree, disagree, neutral, agree, strongly agree) to questions assessing their perceptions of the impacts of COVID-19, their vulnerability to COVID-19, the value of handwashing in preventing COVID-19, and the value of physical distancing in preventing COVID-19 (detailed in Table 2). The Likert scale responses were included in the analysis on a binary scale, comparing those that responded “strongly disagree”, “disagree”, and “neutral” to those that responded “agree” and “strongly agree.” The enumerator indicated in the survey if the respondent, at any point in the survey, indicated that they did not believe COVID-19 exists. Respondents were also asked to indicate if they had more, less, or the same amount of concern about the risks of COVID-19 relative to other prevalent diseases including diarrhea, malaria, cholera, HIV/AIDs, and tuberculosis. Responses to these questions were included in the analysis on a binary scale (more concerned vs. less concerned or the same amount of concern), to assess the impacts of risks perceptions compared to other diseases. For the remainder of this paper, we will refer to all perceptions of risk and health behaviors as “risk perceptions” for simplicity.

#### 2.3.4. COVID-19 preventive behaviors

Outcome variables of interest were self-reported behaviors of handwashing and physical distancing. Handwashing was categorized (yes/no) based on responses to the question “In the last 24 hours, have you washed your hands with soap and water after going to a public place, or after nose-blowing, coughing, or sneezing?”. Physical distancing was categorized based on responses to the question “Are you able to practice social distancing during the COVID-19 pandemic?” We included the self-reported behavior of mask wearing (*Did you wear a mask the last time you went out in public*?) as a behavior we did not expect to be explained by WASH-related factors. Responses to these outcome measures were all analyzed on a binary scale (yes/no). If respondents indicated that they did not practice handwashing or physical distancing, a follow-up question asked about the reasons for not practicing these behaviors.

### 2.4. Statistical analyses

#### 2.4.1. Analysis of factors influencing COVID-19 prevention behaviors

**Fig 2:**
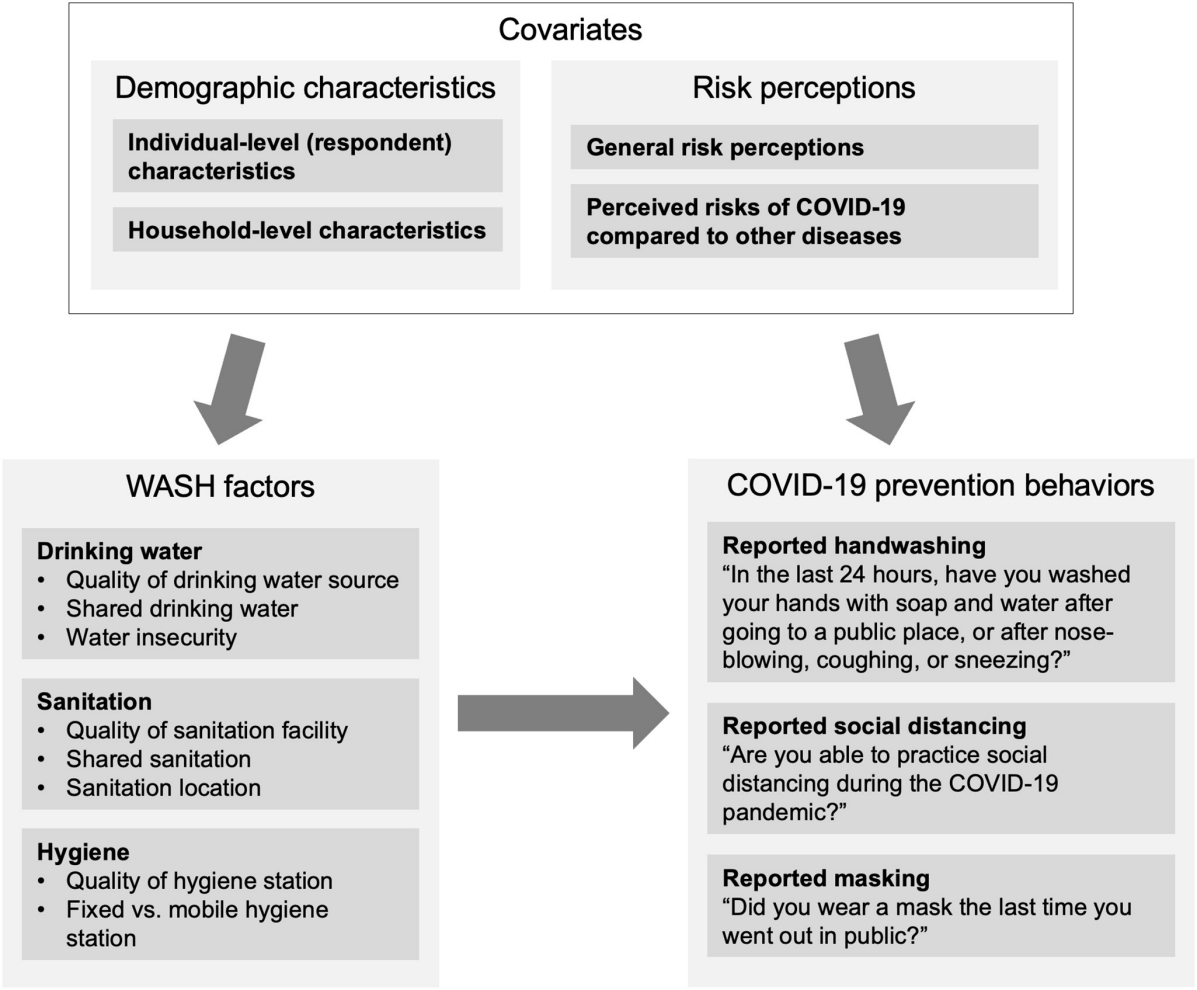
Theory of change for regression models.

Data on demographic factors, risk perceptions, and WASH factors collected during the 2020 survey were used to assess factors that influenced COVID-19 prevention behaviors. The theory of change in Fig 2 summarizes the relationships between variables analyzed in the regression models. To evaluate associations between WASH factors and the three COVID-19 preventative behaviors of interest, adjusting for all demographic characteristics and risk perceptions, we developed unadjusted and fully adjusted logistic regression models to calculate odds ratios (ORs) and 95% confidence intervals. We developed three separate models to explore these relationships–one for each COVID-19 preventative behavior outcome. To address the inflated risk for type I error due to multiple comparisons, we adjusted p-values for false discovery rate within each category of independent variable (demographic characteristics, risk perceptions, and WASH factors) using the Benjamini-Hochberg method [41]. Multicollinearity was assessed for all models and no concerns for multicollinearity of variables were identified. Responses to follow-up questions about why participants did not practice handwashing and physical distancing were also summarized (*n* and %). All analyses were conducted using R version 4.0.0 [42].

### 2.5. Ethics statement

The study was approved by the Mozambique National Bio-Ethics Committee for Health (Ref: 105/CNBS/20) and the Institutional Review Board of Emory University (IRB#: CR001-IRB00098584, Atlanta, GA). We also obtained permissions from local authorities, namely Beira municipality and municipal district administrations from study neighborhoods included in the study. Credential letters were issued to be presented in all sub-neighborhoods and households visited. Additionally, courtesy meetings between the study team and city health department were held. Recruitment and consent of the primary caregiver of children under 18 years of age took place at the households. The recruitment period took place between November 16 to December 23, 2019 for the first survey round and November 7 to December 23, 2020 for the second survey round. Prior to enrollment, study staff fully explained and carried out the consent process and documented the procedure. Parents or guardians of children under 18 years old provided a signature to indicate consent. In the case of illiteracy of the subject, study staff verbally summarized the material with the subject, and the participants were required to mark the document with a thumbprint indicating their consent.

COVID-19 safety protocols were implemented during data collection for this study aligning with the Emory University IRB COVID-19 Guidelines for In-Person Human Subjects Research to protect field workers and study participants (*Emory IRB Guidelines*: *Resuming Non-Essential In-Person Human Subjects Research*, 2020). Specifically, field staff were instructed to conduct surveys outside, participate in physical distancing, wear a mask, and use alcohol-based hand sanitizers while conducting surveys as well as to actively monitor their temperature and avoid interacting with study participants if they were sick. Two questions were included at the beginning of the survey to screen participants for risk of COVID-19, including (1) if they had any symptoms of COVID-19 and (2) if they lived or have recently visited with someone with COVID-19. If participants failed the COVID-19 screening, the survey was terminated, and they were visited at a later time.

## 3. Results

### 3.1. Summary of study population

A total of 1,762 households were surveyed in the 2020 population-based survey (Table 1). Respondents were predominantly female and mothers, and most had either primary or higher education. The largest proportion of respondents lived in households with 4–7 people and about half had children under 5 years old living in the household.

**Table 1:** Demographic characteristics of study population (N = 1762); Beira, Mozambique 2020.

	n (%)
Female respondent	1324 (75.1%)
**Primary caregiver education**	
No or incomplete primary	195 (14.1%)
Primary education	495 (35.8%)
Higher education	694 (50.1%)
**Wealth index**	
1 (poorest)	319 (18.1%)
2	518 (29.4%)
3	539 (30.6%)
4	360 (20.4%)
5 (least poor)	24 (1.4%)
**Household members**	
Less than or equal to 3	329 (18.7%)
Between 4 and 7	1093 (62.0%)
More than 7	340 (19.3%)
Child under-five in household	968 (54.9%)
Respondent is mother	1040 (73.5%)
Pregnant woman in household	189 (10.7%)

### 3.2. Factors influencing COVID-19 prevention behaviors

Summary statistics for the risk perception and WASH factor variables considered in the assessment of factors influencing COVID-19 behaviors are shown in Table 2. The study population generally had a high level of water access, with approximately 98.4% or participants having access to an improved water source. A summary of the 4-question HWISE scale showed that 63.6% of respondents were considered water secure (*not* water insecure). Sanitation access was also high, with 80.8% having access to an improved sanitation facility and 93.5% having a sanitation facility in their yard or dwelling. Handwashing access was relatively low compared to reported rates of drinking water and sanitation access. Most respondents had either a limited handwashing station (21.9%) or no handwashing station (41.8%), and, of those that had a handwashing station, 81.3% had an unconnected handwashing station, or a handwashing station that was not permanently connected to a water source such as a bucket, jug, or kettle.

**Table 2:** Risk perceptions and WASH factors for the study population during the 2020 survey, used for regression analyses (N = 1,762); Beira, Mozambique 2020.

Variable	N	n (%)
Wash hands with soap and water after going to a public place, nose-blowing, coughing, or sneezing	1762	1539 (95.5)
Practice physical distancing during the COVID-19 pandemic	1762	1478 (91.7)
Wear a mask the last time out in public	1762	1492 (92.6)
**Risk Perceptions**		
**“Contracting COVID-19 would have a great impact on your daily life”** (Perceived impact)	1,762	
Neutral/disagree/strongly disagree		192 (10.9)
Agree		632 (35.9)
Strongly agree		938 (53.2)
**“You perceive yourself to be vulnerable to COVID-19”** (Perceived vulnerability)	1,762	
Neutral/disagree/strongly disagree		28 (1.6)
Agree		677 (38.4)
Strongly agree		1057 (60.0)
**“Washing hands is essential to protect myself from COVID-19”** (Importance of handwashing)	1,760	
Neutral/disagree/strongly disagree		50 (2.8)
Agree		760 (43.2)
Strongly agree		950 (54.0)
**“It is important to keep your distance from others to avoid spreading the coronavirus”** (Importance of physical distancing)	1,761	
Neutral/disagree/strongly disagree		410 (23.3)
Agree		575 (32.7)
Strongly agree		776 (44.1)
Respondent indicated they do not believe COVID-19 exists		52 (3.0)
**More concerned about this disease compared to COVID-19**	1762	
diarrhea		214 (12.1)
malaria		199 (11.3)
cholera		301 (17.1)
HIV/AIDs		174 (9.9)
tuberculosis		180 (10.2)
**WASH factors**		
**Drinking water access**		
Improved water source	1762	1733 (98.4)
Personal (not shared) water source^1^	1086	692 (39.3)
Water source in dwelling	1762	1054 (59.8)
Water secure (HWISE < 4)^2^		1118 (63.6)
**Sanitation facility**	1759	
No facility		40 (2.3)
Unimproved		298 (16.9)
Improved		1421 (80.8)
Sanitation facility in dwelling/yard	1762	1648 (93.5)
Personal (not shared) sanitation facility	1760	1140 (64.8)
**Handwashing facility**	1759	
No facility		736 (41.8)
Limited		385 (21.9)
Basic		638 (36.3)
**Handwashing facility connection status**	1023	
Unconnected		832 (81.3)
Connected		191 (18.7)

^1^The question about water sharing were only asked for people that had a connection to a water service line from the water utility. This variable was not included in regression analyses because of the high level of missingness and because it was only asked for a specific subset of respondents.

^2^Scores for the HWISE scale range from 0–12, where higher scores indicate higher levels of water insecurity (Median = 2.00, SD = 2.93).

Respondents were highly engaged in COVID-19 preventative behaviors, including handwashing (95.5%), physical distancing (91.7%), and mask wearing (92.6%) (Table 2). Most respondents indicated that they agreed or strongly agreed with all risk perception statements, indicating that they held beliefs that are likely to facilitate positive COVID-19 preventative behaviors. Only 3.0% of respondents indicated that they did not believe in COVID-19. However, many respondents reported being more concerned about other diseases than COVID-19, ranging from 9.9% for HIV to 17.1% for cholera.

**Fig 3:**
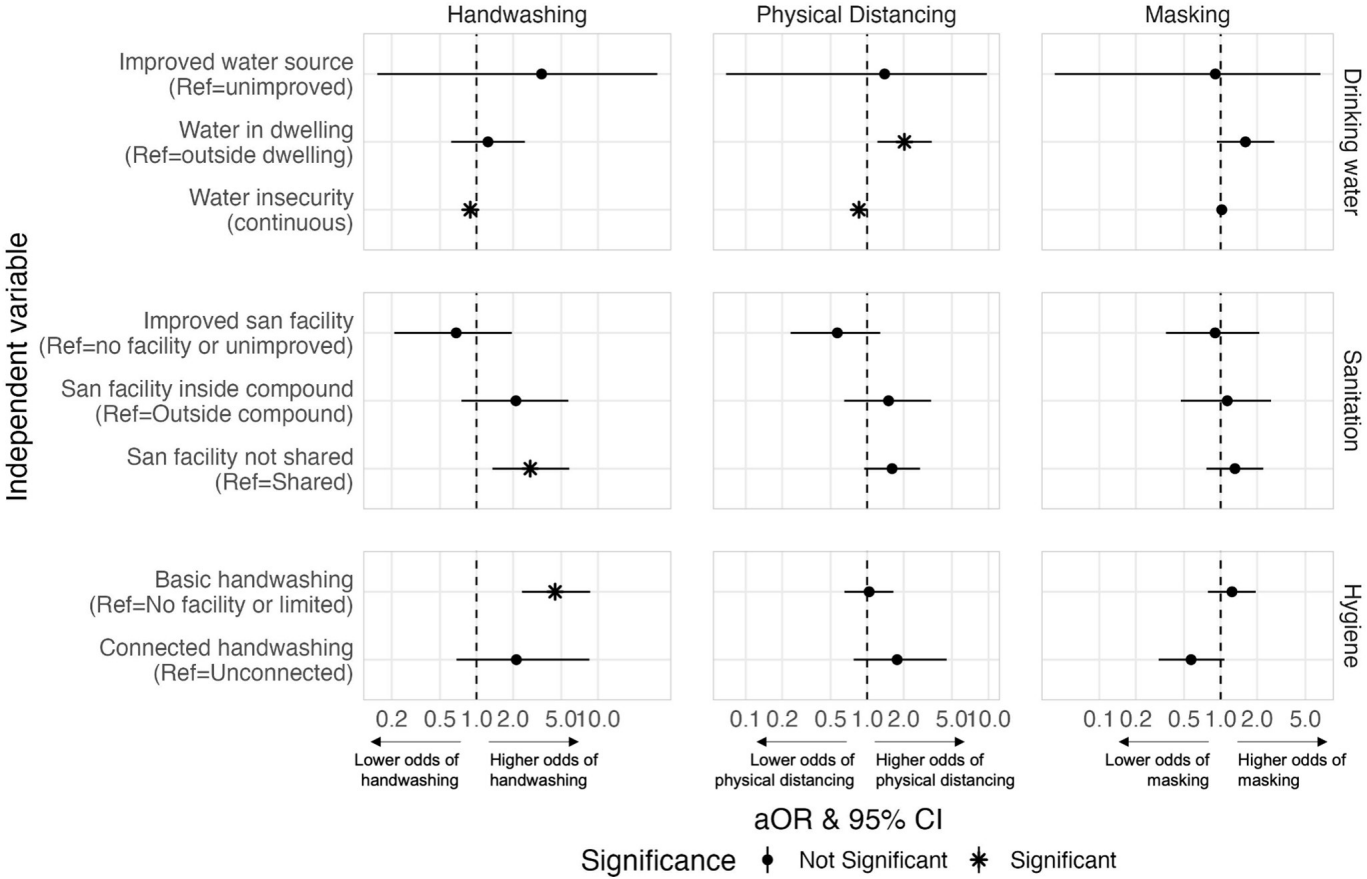
Adjusted associations between WASH factors and self-reported COVID-19 preventative behaviors. Odds ratios are adjusted for all other variables in the model (all demographic characteristics, risk perceptions and other WASH factors). Correction for multiple testing was performed, and therefore statistical significance is based on an α of 0.05 while also adjusting for false discovery rate. (Beira, Mozambique 2020).

**Fig 4:**
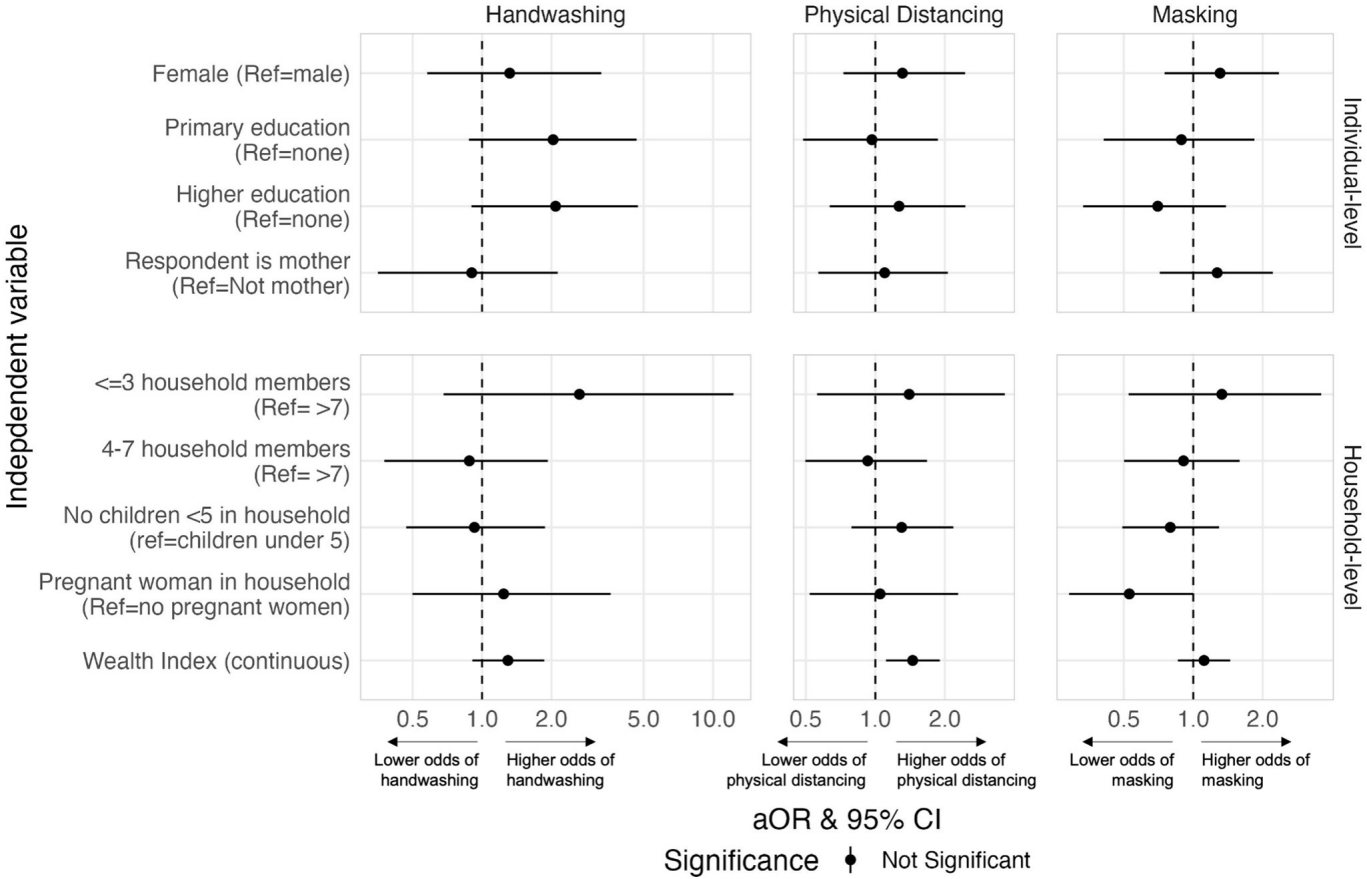
Adjusted associations between individual- and household-level demographic characteristics and self-reported COVID-19 preventative behaviors. Odds ratios are adjusted for all other variables in the model (all risk perceptions, WASH factors, and other demographic characteristics). Correction for multiple testing was performed, and therefore statistical significance is based on an α of 0.05 while also adjusting for false discovery rate. (Beira, Mozambique 2020).

**Fig 5:**
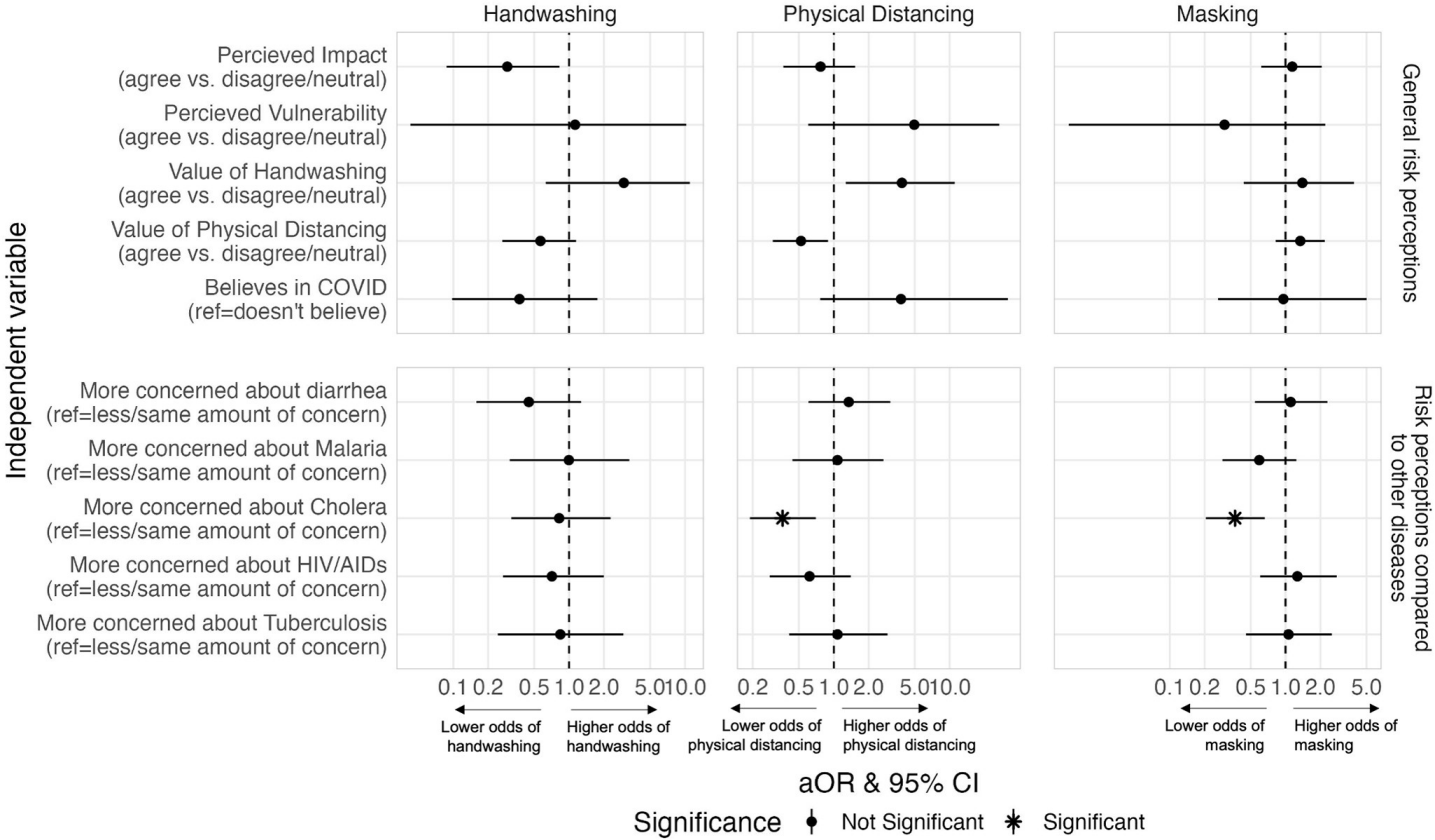
Adjusted associations between risk perceptions and self-reported COVID-19 preventative behaviors. Odds ratios are adjusted for all other variables in the model (all demographic characteristics, WASH factors, and other risk perceptions). Correction for multiple testing was performed, and therefore statistical significance is based on an α of 0.05 while also adjusting for false discovery rate. (Beira, Mozambique 2020).

Results from the adjusted regression analyses assessing relationships between hypothesized determinants of behavior and COVID-19 preventative behaviors are shown in Figs 3–5 (full data presented in S3 Table). Several WASH factors were found to be associated with handwashing and physical distancing (Fig 3). No strong relationships between WASH factors and reported masking were identified in the adjusted analysis. Having higher levels of water insecurity was associated with a lower odds of handwashing [aOR = 0.89; 95% CI: 0.81, 0.98] and a lower odds of physical distancing [aOR = 0.85; 95% CI: 0.80, 0.92]. Having a water source within one’s dwelling, compared to having a water source outside one’s dwelling, was associated with a higher odds of physical distancing [aOR = 2.03; 95% CI: 1.22, 3.41]. Several indicators of sanitation security were associated with a higher odds of handwashing and physical distancing: having a sanitation facility inside of one’s compound, compared to outside one’s compound [handwashing aOR = 2.11; 95% CI: 0.75, 5.71; distancing aOR = 1.50; 95% CI: 0.65, 3.36], and having a personal sanitation facility, compared a shared facility [handwashing aOR = 2.77; 95% CI: 1.35, 5.82; distancing aOR = 1.61; 95% CI: 0.95, 2.73]. Indicators of handwashing security were associated with higher odds of handwashing: having a basic handwashing facility, compared to a limited facility or no facility [handwashing aOR = 4.45; 95% CI: 2.37, 8.65], and having a connected handwashing station, compared to an unconnected handwashing station [handwashing aOR = 2.13; 95% CI: 0.68, 8.54]. Having a connected handwashing station was also positively associated with physical distancing [aOR = 1.77; 95% CI: 0.77, 4.53].

Demographic characteristics (Fig 4) and risk perceptions (Fig 5) also appear to influence decisions to practice disease prevention behaviors, based on findings from our adjusted regression analyses (see S3 Table for all aORs and 95% CIs). Higher levels of education for the primary caregiver was associated with a higher odds of handwashing [aOR = 2.03; 95% CI: 0.88, 4.66 for primary education; aOR = 2.08; 95% CI: 0.90, 4.73 for higher education]. Less dense households had a higher odds of reporting all COVID-19 prevention behaviors and having higher wealth was associated with a higher odds of reported handwashing [aOR = 1.29; 95% CI: 0.91, 1.86] and a higher odds of physical distancing [aOR = 1.45; 95% CI: 1.11, 1.91]. Believing in the value of handwashing for preventing transmission COVID-19 was associated with a higher odds of handwashing [aOR = 2.97; 95% CI: 0.63, 11.0] and physical distancing [aOR = 3.87; 95% CI: 1.27, 11.1]. On the other hand, believing in the value of physical distancing was associated with a lower odds of handwashing [aOR = 0.57; 95% CI: 0.27, 1.15] and physical distancing [aOR = 0.52; 95% CI: 0.30, 0.89]. Finally, being more concerned about cholera than COVID-19 was associated with a lower odds of physical distancing [aOR = 0.36; 95% CI: 0.19, 0.70] and a lower odds of masking [aOR = 0.37; 95% CI: 0.20, 0.66].

**Fig 6:**
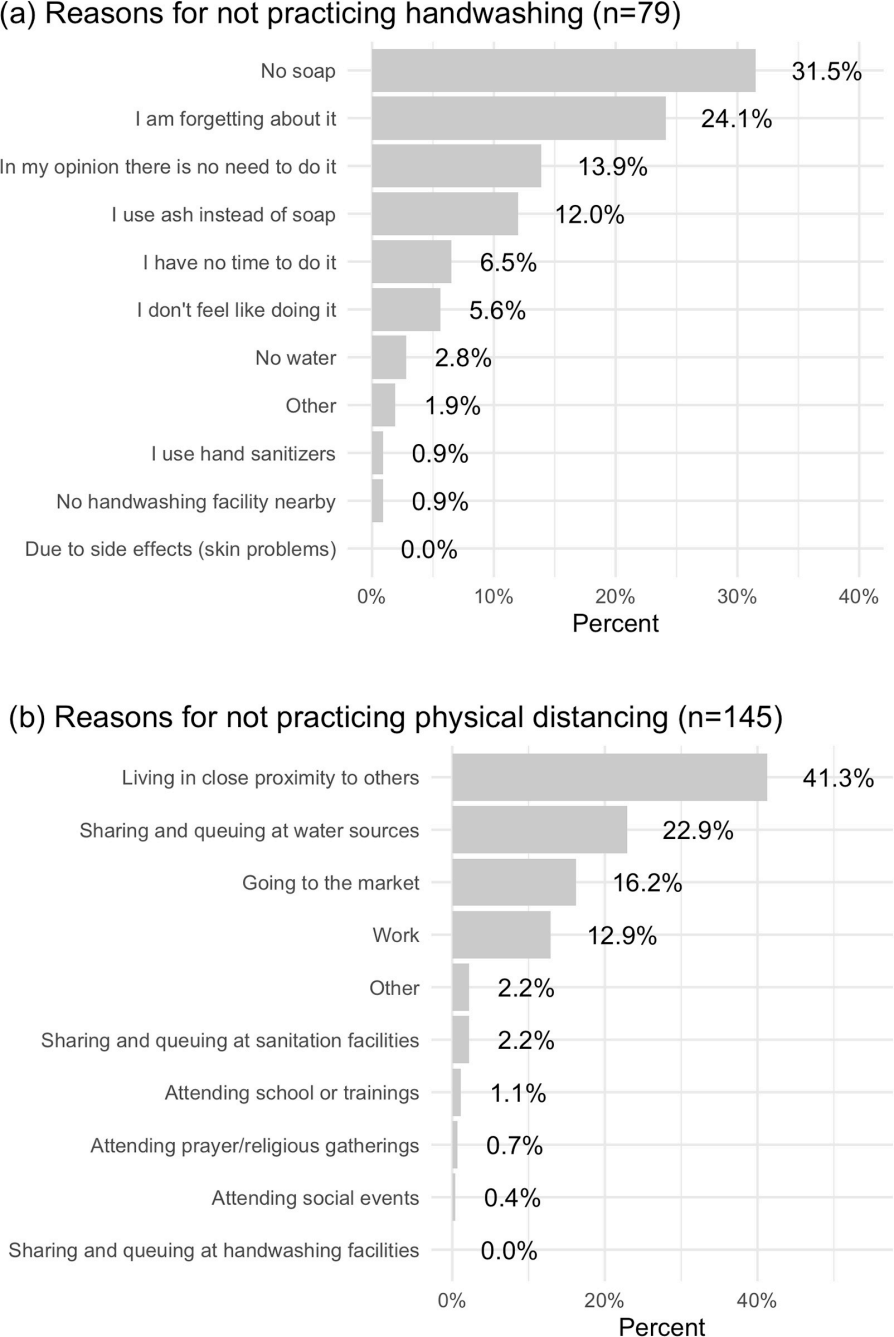
Reported reasons for not practicing (a) handwashing and (b) physical distancing; Beira, Mozambique 2020.

Reported reasons for not practicing handwashing and physical distancing are summarized in Fig 6. The most commonly reported reasons for not practicing handwashing were centered around having poor quality handwashing stations (no soap, using ash instead of soap, no water) and not believing in the importance of handwashing (forgetting about it, feeling no need to do it, not feeling like doing it). The most commonly reported reasons for not practicing physical distancing were living in close proximity to others and participating in daily activities around others (sharing and queuing at water sources, going to the market, work).

## 4. Discussion

Our survey data from low-income neighborhoods of Beira, Mozambique led to several interesting insights on factors that influenced people’s abilities and decisions to practice COVID-19 prevention measures. Demographic characteristics and risk perceptions played an important role in influencing people’s disease prevention behaviors, but WASH access appeared to be a particularly critical determinant of behavior given the strength and magnitude of the effect estimates compared to other determinants considered. Even with a high level of knowledge about the risks of COVID-19 and benefits of practicing handwashing and physical distancing, WASH insecurity acted as a key barrier preventing people from practicing these behaviors in response to the COVID-19 pandemic. Our findings highlight the importance of WASH infrastructure in building resilience to future health crises.

### 4.1. WASH access factors

WASH insecurity influenced decisions to practice handwashing and physical distancing during the pandemic. Specifically, reported handwashing and physical distancing was lower among respondents who had higher levels of water insecurity. People facing water insecurity, or those that have to go outside their homes to access water, may be unable to wash their hands or practice other personal hygiene habits [4, 5]. Sanitation infrastructure also influenced respondent’s decisions. Having a shared sanitation facility and having to use a sanitation facility outside of one’s compound was also associated with lower odds of reported handwashing and physical distancing. This supports evidence that highlights the increased risk of transmission of COVID-19 for people that use a shared sanitation facility due to their increased interactions with others [5, 27, 43]. Finally, reported handwashing was higher for people that had higher quality handwashing infrastructure, including a basic handwashing station and a fixed handwashing station. While the quality of handwashing stations has been identified as a key factor influencing hygiene decisions during non-pandemic conditions [8, 27], here we show the importance of handwashing infrastructure in enabling people to practice handwashing during an active health crisis. These findings provide evidence that WASH infrastructure plays an important role in influencing the ability and decisions to practice important disease prevention behaviors.

Mozambique had a strong early response to the COVID-19 pandemic, with governmental guidance for handwashing and physical distancing and strong reported adherence to safety measures [31]. Most respondents in this study held beliefs that would act as motivators to practicing handwashing and physical distancing. Even with this high level of awareness about the importance of practicing disease prevention behaviors, WASH insecurity acted as a barrier to practicing physical distancing and handwashing. While there may have been a slight improvement in the availability of water in 2020 for people in the study area (S1 Table), people likely needed more water during the pandemic due to the higher perceived value of handwashing. This is further supported by reported barriers to practicing handwashing and physical distancing (Fig 6). Having no soap and using ash instead of soap were some of the most commonly reported barriers to practicing handwashing and sharing and queuing at water sources and sanitation facilities were commonly reported barriers to practicing physical distancing. While behavioral messaging around the importance of disease prevention behaviors is necessary to promote handwashing and physical distancing, without access to adequate WASH infrastructure, people may not be able to practice behaviors necessary for reducing transmission of disease.

### 4.2. Demographic and perception factors

Our analyses highlighted the role of some demographic characteristics in influencing decisions to practice COVID-19 prevention behaviors. For example, while physical distancing was widely promoted as a disease prevention measure, people may be unable to consistently physically distance due to the need to attend work [26, 27, 44, 45]. This was echoed by reports that “going to the market” and “work” were common barriers to physical distancing (Fig 6b). Household characteristics, including wealth and household size were also found to play a role in decisions (i.e. people with more wealth had a higher odds of reporting physical distancing and handwashing and people with larger house sizes had a lower odds of handwashing and physical distancing). This study supports evidence from several other studies that have shown the importance of some household characteristics, especially factors associated with socio-economic status, in influencing disease prevention behaviors [46–48].

Risk perceptions may also play an important role in people’s decisions to practices disease prevention behaviors. For example, reported handwashing and physical distancing was higher among people that believed in the value of handwashing for preventing the transmission of COVID-19. Some results regarding risk perceptions were mixed and went against expectations regarding facilitators of behavior. For example, people that indicated that they strongly agree or agree that there is value in physical distancing reported less physical distancing. This may indicate bias in reporting, as has been identified as a concern in other studies [49, 50], or it may indicate that other factors may overpower perceptions of risk when making decisions around disease prevention behaviors–i.e., while people may believe in the value of physical distancing, economic factors or WASH insecurity may overcome the influence of those perceptions to determine people’s behaviors.

### 4.3. Limitations

Data collection for this study occurred around the time of several compounding health crises in the area, including Cyclone Idai and a large cholera outbreak, on top of COVID-19 [26]. Along with these health crises, the study area experienced important shifts in WASH infrastructure, both due to damage from the cyclone and an increase in investments to improve the infrastructure in the area (e.g., World Bank funding for improvements to the water supply). There was an overall improvement in access and satisfaction with water sources from 2019 to 2020 (S1 Table), although there is heterogeneity of these characteristics across different areas of the study population [35]. Within this context, risk perceptions about COVID-19 may have been influenced by the combination of factors leading up to the pandemic. For example, people that reported being more concerned about cholera than COVID-19 had a lower odds of reported physical distancing. While COVID-19 was an active concern for people during the time of the second data collection period for this study, the role of these other health crises in influencing decisions may be more complex. This analysis is, therefore, limited in the conclusions it can draw about the specific impacts of these multiple converging health emergencies on infrastructure and WASH behaviors, particularly the role of Cyclone Idai, because no matching data was collected prior to 2019. These results may not be indicative of behaviors during more “normal” conditions. Mozambique is likely to experience more climate-change related events as well as other health crises in the future [26, 51, 52], so it is important to understand the complex role these external factors play in influencing behavior.

The discussion of the analysis assessing relationships between WASH factors and disease prevention behaviors relies on the interpretation of both “significant” and “non-significant” findings (based on α = 0.05). While scientific research often relies heavily on the use of p-values, p-values alone cannot act as a good measure of evidence when interpreting models [53]. Non-significant findings can still provide important insight into the data, particularly when assessing multiple comparisons as is done in this analysis. In the discussion, we highlight important relationships found in the models, as reflected by the magnitude and direction of the effect estimates rather than just “statistical significance”.

Finally, several of the variables used in these analyses, including the primary outcome measure of reported disease prevention behaviors, are based on respondent reports and therefore may be prone to recall bias and over reporting. Additionally, the reported disease prevention behaviors only asked about behaviors in the 24 hours prior to the survey. It is possible that people’s behaviors within the recall period do not align with their long-term practices and may bias results. For other reported variables, where possible, standard metrics were used and adjustments to regression models were made to avoid influence of bias.

## 5. Conclusion

While the acute phase of the COVID-19 pandemic may be over, there is much we can learn about how to manage future health emergencies. Results from our analysis highlight the important role of WASH access in enabling people to practice disease prevention behaviors in response to the COVID-19 pandemic. While demographic factors and risk perceptions, including wealth and beliefs about the value of handwashing and physical distancing, influenced decisions to practice disease prevention behaviors, WASH access was found to be the most important determinant of behavior. Despite the high level of knowledge about the value of handwashing and physical distancing, inadequate WASH infrastructure, including water insecurity, having to leave one’s house for drinking water, the use of shared sanitation, and poor quality handwashing stations, was a key barrier to practicing those behaviors. We add to previous literature analyzing determinants of handwashing and physical distancing decisions under pandemic conditions by specifically exploring WASH access as a determinant of behavior. Our analysis suggests that limitations in WASH infrastructure acted as a key limiting factor for people in practicing hygiene behaviors during the pandemic. Future research should focus on optimal ways to provide WASH services to people with insufficient access, to enable populations to respond to future health crises. These findings are valuable for understanding motivators of disease prevention behaviors and could help inform future guidance on WASH for pandemic response.

## Supporting information

S1 Table Longitudinal changes in facets of water insecurity for pre- and under pandemic conditions. (DOCX)

S2 Table Reported changes in water provisions. (DOCX)

S3 Table Full results from adjusted logistic regression models assessing associations between hypothesized determinants of behavior (demographic characteristics, WASH factors, and risk perceptions) and COVID-19 preventative behaviors (handwashing, social distancing, and masking). (DOCX)
