# Supplementary material for: Water, sanitation, and hygiene insecurity and disease prevention behaviors during the COVID-19 pandemic in low-income neighborhoods of Beira, Mozambique
Source: PLoS One. 2024 Nov 21;19(11):e0310490. doi: 10.1371/journal.pone.0310490 (PMC11581246; doi:10.1371/journal.pone.0310490)
Supplement: S1 Table — (DOCX) [file pone.0310490.s001.docx]

**Methods for analysis of longitudinal changes in water and sanitation access**

**Additional water access variables for S1**

Supplementary analyses considered additional facets of water insecurity including availability, accessibility, affordability, and satisfaction. Accessibility was evaluated based on the proportion of households that used an improved drinking water source, the proportion of households that had an improved, on-site drinking water source, and the proportion of households that used the same water source for drinking and other purposes. Water availability was evaluated using the reported number of hours per day and the number of days per week that water was available as well as the proportion of households reporting insufficient quantities of drinking water in the last month. Respondents were asked if they spent more than 150 MT (approximately $2.30 in 2019, considered to be typical monthly expense for water by local study partners) on drinking water in the past month (yes/no), which was used to assess water affordability. Respondents were asked to report how often (never/sometimes/always) they were satisfied with overall service, water pressure, and water quality of their main source of drinking water. The responses to the water satisfaction questions related to services, pressure, and quality were recoded as binary, comparing those who were sometimes or always satisfied with their water provision (yes) to those who were never satisfied (no).

Two additional survey questions were asked during both surveys about perceived changes in water sources in the past year. Respondents were first asked if there were positive changes in water quality, water pressure, number of hours of supply, and the number of outages, then they were asked if there were negative changes in those drinking water characteristics.

**Statistical methods for analyzing longitudinal changes in water and sanitation access for S1**

Data on facets of WASH access were analyzed to understand the conditions of water and sanitation access within the study population and the extent to which facets of WASH insecurity changed before and after the start of the COVID-19 pandemic. We calculated summary statistics to observe proportional changes in facets of water insecurity between the 2019 and 2020 surveys. Facets of water insecurity included water access, water availability, water affordability, and satisfaction with water provisions. Changes in sanitation access were evaluated based on JMP definitions of sanitation quality, whether the facility was shared, and the location of the sanitation facility.

All of the sub-neighborhoods visited in the 2019 survey were also surveyed during the 2020 survey, and two additional neighborhoods were added during the 2020 survey; these added sub-neighborhoods were excluded from the longitudinal analysis. After excluding households in neighborhoods not visited during the 2019 survey, there were 1626 households from the 2019 survey and 1612 households from the 2020 survey included in the longitudinal analysis shown in S1. Responses to perceived changes in drinking water within the year prior to both surveys are summarized in S2.

| **Indicator** | **2019 pre-pandemic conditions**  **(*N*=1626)** | **2020 pandemic conditions**  **(*N*=1612)** | **Percent Difference**  (95% CI) |
| --- | --- | --- | --- |
| **Water Access** |  |  |  |
| Household (HH) drinking water from an improved water source, *N* (%)* | 1595 (98.1%) | 1602 (99.4%) | +1.3% (0.5%, 2.1%) |
| HH drinking water from an improved water source that is located on premises, *N* (%) | 767 (47.2%) | 982 (60.9%) | +13.7% (10.3%, 17.1%) |
| Personal (not shared) water source, of those that had a water connection to their HH^1^, *N* (%)* | 612 (67.3%) | 656 (63.8%) | - 3.5% (-0.8%, 7.9%) |
| HH uses the same water source for drinking and other purposes, such as cooking and hand washing, *N* (%) | 1407 (86.5%) | 1450 (90.0%) | +3.5% (1.2%, 5.6%) |
| **Water Availability** |  |  |  |
| Water is available from main source of drinking water, hours per day | 10.71 | 12.45 | +1.74 (1.33, 2.14) |
| Water is available from main source of drinking water, days per week | 6.29 | 6.16 | -0.13 (-0.23, -0.03) |
| Reported insufficient quantities of drinking water any time in the last month, *N* (%) | 425 (26.1%) | 540 (33.5%) | +7.4% (4.2%, 10.5%) |
| **Water Affordability** |  |  |  |
| Spent > 150 MT on water per month, *N* (%) | 1275 (78.4%) | 1241 (77.0%) | -1.4% (-4%, 10.7%) |
| **Water Satisfaction** |  |  |  |
| Always satisfied with the service of drinking water, *N* (%) | 329 (20.2%) | 723 (44.9%) | +24.7% (21.5%, 27.7%) |
| Always satisfied with the pressure of drinking water, *N* (%) | 499 (31.3%) | 861 (53.7%) | +22.4% (19.1%, 25.8%) |
| Always satisfied with the quality of drinking water, *N* (%) | 176 (10.8%) | 428 (26.6%) | +15.8% (13.1%, 18.4%) |
| **Sanitation Access** |  |  |  |
| Improved sanitation facility, *N* (%)* | 1495 (92.0%) | 1321 (82.0%) | -10.0% (-12.3%, -7.6%) |
| Sanitation facility in dwelling/yard, *N* (%)* | 1443 (88.8%) | 1516 (94.0%) | +5.2% (3.3%, 7.2%) |
| Personal (not shared) sanitation facility, *N* (%)* | 913 (56.2%) | 1043 (64.7%) | +8.5% (5.1%, 12.0%) |

*These variables are included in both S1 Table 1 and Table 2 in the main text. Values across tables are slightly different for these variables because the S1 Table only includes data from neighborhoods visited during both surveys whereas data in Table 2 includes the two neighborhoods added for the 2020 survey.

^1^Water sharing was only assessed for those that had a piped water connection to their household - N=909 in 2019; 1028 in 2020
