# Supplementary material for: Water, sanitation, and hygiene insecurity and disease prevention behaviors during the COVID-19 pandemic in low-income neighborhoods of Beira, Mozambique
Source: PLoS One. 2024 Nov 21;19(11):e0310490. doi: 10.1371/journal.pone.0310490 (PMC11581246; doi:10.1371/journal.pone.0310490)
Supplement: S2 Table — (DOCX) [file pone.0310490.s002.docx]

|  | **Responded ‘yes’ - Pre-pandemic conditions**  n (%) | **Responded ‘yes’ – Under pandemic conditions**  n (%) | **Change**  % (95% CI) |
| --- | --- | --- | --- |
| Has your water service changed in the past year? | 473 (29.1) | 371 (23.1) | -6.1% (-9.1%, -3.1%) |
| **Positive changes**  “Has your ___ improved in the past year?” | |  |  |
| Water quality | 90 (19.0) | 168 (45.3) | 26.3% (20.1%, 32.5%) |
| Water pressure | 97 (20.5) | 190 (51.2) | 30.7% (24.4%, 37%) |
| Number of hours of supply | 85 (18.0) | 186 (50.1) | 32.1% (26%, 38.3%) |
| Number of outages | 83 (17.5) | 178 (48.0) | 30.5% (24.3%, 36.6%) |
| **Negative changes**  “Has your ___ gotten worse in the past year?” | |  |  |
| Water quality | 300 (63.4) | 141 (38.0) | -25.4% (-32%, -18.8%) |
| Water pressure | 216 (45.7) | 134 (36.1) | -9.6% (-16.2%, -2.9%) |
| Number of hours of supply | 254 (53.7) | 129 (34.8) | -18.9% (-25.6%, -12.3%) |
| Number of outages | 230 (48.6) | 130 (35.0) | -13.6% (-20.2%, -6.9%) |
