# Supplementary material for: Water, sanitation, and hygiene insecurity and disease prevention behaviors during the COVID-19 pandemic in low-income neighborhoods of Beira, Mozambique
Source: PLoS One. 2024 Nov 21;19(11):e0310490. doi: 10.1371/journal.pone.0310490 (PMC11581246; doi:10.1371/journal.pone.0310490)
Supplement: S3 Table — (DOCX) [file pone.0310490.s003.docx]

|  | **Variable category** | **Level** | **Handwashing** | | **Social Distancing** | | **Masking** | |
| --- | --- | --- | --- | --- | --- | --- | --- | --- |
|  |  |  | **Odds Ratio**  **(95% CI)** | **p-value** | **Odds Ratio**  **(95% CI)** | **p-value** | **Odds Ratio**  **(95% CI)** | **p-value** |
| **Demographic Factors** | | |  |  |  |  |  |  |
| Individual-level (respondent) characteristics | |  |  |  |  |  |  |  |
|  | Female (ref = Male) |  | 1.32 (0.58, 3.28) | 0.531 | 1.31 (0.73, 2.45) | 0.380 | 1.31 (0.75, 2.35) | 0.356 |
|  | Respondent Education  (Ref = No or incomplete primary) | Primary education | 2.03 (0.88, 4.66) | 0.094 | 0.97 (0.49, 1.87) | 0.921 | 0.89 (0.41, 1.84) | 0.756 |
|  |  | Higher education | 2.08 (0.9, 4.73) | 0.081 | 1.27 (0.63, 2.46) | 0.491 | 0.70 (0.33, 1.39) | 0.327 |
|  | Respondent is a mother (ref= respondent is not a mother) |  | 0.9 (0.35, 2.13) | 0.819 | 1.10 (0.57, 2.06) | 0.776 | 1.27 (0.72, 2.21) | 0.406 |
| Household-level characteristics | |  |  |  |  |  |  |  |
|  | Wealth quintile (cont.) |  | 1.29 (0.91, 1.86) | 0.159 | 1.45 (1.11, 1.91) | 0.006 | 1.11 (0.86, 1.45) | 0.420 |
|  | Household members (ref = Less than or equal to 3 people) | Between 4 and 7 people | 0.33 (0.09, 1.00) | 0.074 | 0.66 (0.30, 1.36) | 0.281 | 0.68 (0.29, 1.46) | 0.349 |
|  |  | More than 7 people | 0.38 (0.08, 1.47) | 0.180 | 0.71 (0.27, 1.79) | 0.477 | 0.75 (0.28, 1.91) | 0.558 |
|  | No child under five in household (ref = child under 5 in household) |  | 0.93 (0.47, 1.87) | 0.830 | 1.30 (0.79, 2.18) | 0.309 | 0.80 (0.49, 1.29) | 0.352 |
|  | Pregnant woman in household (ref = pregnant woman not in household) |  | 1.24 (0.50, 3.60) | 0.665 | 1.05 (0.52, 2.29) | 0.898 | 0.53 (0.29, 1.00) | 0.043 |
| **WASH Factors** | | |  |  |  |  |  |  |
| Drinking water source | |  |  |  |  |  |  |  |
|  | Improved water source (ref = unimproved) |  | 3.44 (0.15, 30.88) | 0.317 | 1.39 (0.07, 9.69) | 0.773 | 0.90 (0.04, 6.66) | 0.932 |
|  | Water in dwelling (ref= not in dwelling) |  | 1.24 (0.62, 2.51) | 0.541 | 2.03 (1.22, 3.41) | 0.007 | 1.60 (0.93, 2.77) | 0.089 |
|  | Water insecurity (cont.; higher values = more water insecure) |  | 0.89 (0.81, 0.98) | 0.017 | 0.85 (0.80, 0.92) | 0.000 | 1.02 (0.94, 1.11) | 0.607 |
| Sanitation facility | |  |  |  |  |  |  |  |
|  | Improved sanitation facility (ref = No facility or unimproved facility) |  | 0.68 (0.21, 1.95) | 0.494 | 0.57 (0.23, 1.28) | 0.190 | 0.90 (0.35, 2.08) | 0.815 |
|  | Sanitation facility inside compound (ref = outside compound) |  | 2.11 (0.75, 5.71) | 0.147 | 1.50 (0.65, 3.36) | 0.332 | 1.13 (0.47, 2.60) | 0.772 |
|  | Sanitation facility not shared (ref = shared) |  | 2.77 (1.35, 5.82) | 0.006 | 1.61 (0.95, 2.73) | 0.079 | 1.31 (0.76, 2.25) | 0.322 |
| Handwashing station | |  |  |  |  |  |  |  |
|  | Basic handwashing facility (ref = no facility or limited facility) |  | 4.45 (2.37, 8.65) | 0.000 | 1.04 (0.65, 1.65) | 0.873 | 1.24 (0.79, 1.95) | 0.351 |
|  | Connected handwashing station (ref = Unconnected) |  | 2.13 (0.68, 8.54) | 0.231 | 1.77 (0.77, 4.53) | 0.202 | 0.57 (0.31, 1.07) | 0.076 |
| **Risk perceptions** | |  |  |  |  |  |  |  |
|  | Contracting COVID-19 would have a great impact on your daily life (ref = neutral/disagree/strongly disagree) | Agree/Strongly agree | 0.29 (0.09, 0.83) | 0.030 | 0.77 (0.37, 1.53) | 0.465 | 1.14 (0.62, 2.05) | 0.665 |
|  | You perceive yourself to be vulnerable to COVID-19 (ref = neutral/disagree/strongly disagree) | Agree/Strongly agree | 1.13 (0.04, 10.25) | 0.928 | 4.95 (0.60, 26.8) | 0.085 | 0.30 (0.01, 2.21) | 0.315 |
|  | Washing hands is essential to protect myself from COVID-19 (ref = neutral/disagree/strongly disagree) | Agree/Strongly agree | 2.97 (0.63, 11.0) | 0.128 | 3.87 (1.27, 11.1) | 0.013 | 1.40 (0.44, 3.90) | 0.545 |
|  | It is important to keep your distance from others, to avoid spreading the coronavirus (ref = neutral/disagree/strongly disagree) | Agree/Strongly agree | 0.57 (0.27, 1.15) | 0.126 | 0.52 (0.30, 0.89) | 0.020 | 1.34 (0.82, 2.18) | 0.239 |
|  | Respondent indicate they do not believe COVID-19 exists (ref = did not indicate this) |  | 0.37 (0.10, 1.76) | 0.173 | 3.80 (0.76, 31.9) | 0.148 | 0.96 (0.26, 5.01) | 0.953 |
|  | More concerned about this disease compared to COVID-19 (ref = less/same amount of concern) | Diarrhea | 0.45 (0.16, 1.27) | 0.129 | 1.35 (0.61, 3.08) | 0.472 | 1.11 (0.54, 2.29) | 0.781 |
|  |  | Malaria | 1.00 (0.31, 3.32) | 0.995 | 1.08 (0.44, 2.68) | 0.875 | 0.59 (0.29, 1.24) | 0.162 |
|  |  | Cholera | 0.82 (0.32, 2.29) | 0.695 | 0.36 (0.19, 0.70) | 0.002 | 0.37 (0.20, 0.66) | 0.001 |
|  |  | HIV/AIDs | 0.71 (0.27, 2.00) | 0.503 | 0.62 (0.28, 1.40) | 0.241 | 1.26 (0.60, 2.76) | 0.545 |
|  |  | Tuberculosis | 0.84 (0.24, 2.94) | 0.785 | 1.08 (0.41, 2.91) | 0.882 | 1.06 (0.46, 2.51) | 0.891 |
